# Supplementary material for: Modeling glioblastoma heterogeneity as a dynamic network of cell states
Source: Mol Syst Biol. 2021 Sep 16;17(9):e10105. doi: 10.15252/msb.202010105 (PMC8444284; doi:10.15252/msb.202010105)
Supplement: Supplementary file 6 — Source Data for Figure 5 [file MSB-17-e10105-s004.zip › Figure5A_sourcedata/GSEA_3071/hallmarks_stateIV.GseaPreranked.1623742551702/HALLMARK_IL2_STAT5_SIGNALING.html]

Details for gene set HALLMARK\_IL2\_STAT5\_SIGNALING[GSEA]

|  || Dataset | state53071 |
| Phenotype | NoPhenotypeAvailable |
| Upregulated in class | na\_neg |
| GeneSet | HALLMARK\_IL2\_STAT5\_SIGNALING |
| Enrichment Score (ES) | -0.47155958 |
| Normalized Enrichment Score (NES) | -2.3645144 |
| Nominal p-value | 0.0 |
| FDR q-value | 0.0 |
| FWER p-Value | 0.0 |
Table: GSEA Results Summary

  

Fig 1: Enrichment plot: HALLMARK\_IL2\_STAT5\_SIGNALING      
 Profile of the Running ES Score & Positions of GeneSet Members on the Rank Ordered List

  

| PROBE | GENE SYMBOL | GENE\_TITLE | RANK IN GENE LIST | RANK METRIC SCORE | RUNNING ES | CORE ENRICHMENT || 1 | SYT11 |  |  | 67 | 1.740 | 0.0125 | No |
| 2 | TNFRSF21 |  |  | 232 | 1.184 | -0.0017 | No |
| 3 | RGS16 |  |  | 237 | 1.178 | 0.0147 | No |
| 4 | SPRY4 |  |  | 304 | 1.030 | 0.0171 | No |
| 5 | GPX4 |  |  | 480 | 0.831 | -0.0044 | No |
| 6 | CDKN1C |  |  | 565 | 0.751 | -0.0096 | No |
| 7 | ITGA6 |  |  | 645 | 0.694 | -0.0147 | No |
| 8 | BMPR2 |  |  | 677 | 0.673 | -0.0108 | No |
| 9 | POU2F1 |  |  | 718 | 0.649 | -0.0090 | No |
| 10 | HIPK2 |  |  | 896 | 0.563 | -0.0348 | No |
| 11 | APLP1 |  |  | 1035 | 0.510 | -0.0539 | No |
| 12 | RORA |  |  | 1060 | 0.503 | -0.0512 | No |
| 13 | CD81 |  |  | 1095 | 0.493 | -0.0505 | No |
| 14 | PRNP |  |  | 1110 | 0.488 | -0.0461 | No |
| 15 | IGF1R |  |  | 1140 | 0.482 | -0.0446 | No |
| 16 | CSF1 |  |  | 1189 | 0.469 | -0.0470 | No |
| 17 | IGF2R |  |  | 1434 | 0.408 | -0.0879 | No |
| 18 | CASP3 |  |  | 1543 | 0.380 | -0.1031 | No |
| 19 | SYNGR2 |  |  | 1577 | 0.371 | -0.1041 | No |
| 20 | DENND5A |  |  | 1673 | 0.355 | -0.1171 | No |
| 21 | ETV4 |  |  | 1937 | 0.307 | -0.1632 | No |
| 22 | CD44 |  |  | 2197 | 0.268 | -0.2090 | No |
| 23 | MAPKAPK2 |  |  | 2534 | -0.273 | -0.2696 | No |
| 24 | SWAP70 |  |  | 2578 | -0.279 | -0.2738 | No |
| 25 | RHOB |  |  | 2638 | -0.288 | -0.2809 | No |
| 26 | GUCY1B1 |  |  | 2642 | -0.288 | -0.2773 | No |
| 27 | AHCY |  |  | 2734 | -0.297 | -0.2905 | No |
| 28 | CCNE1 |  |  | 2741 | -0.299 | -0.2873 | No |
| 29 | NOP2 |  |  | 2746 | -0.300 | -0.2836 | No |
| 30 | BCL2 |  |  | 2901 | -0.319 | -0.3086 | No |
| 31 | FURIN |  |  | 2938 | -0.324 | -0.3108 | No |
| 32 | PTRH2 |  |  | 2961 | -0.327 | -0.3102 | No |
| 33 | PHTF2 |  |  | 2993 | -0.332 | -0.3113 | No |
| 34 | BCL2L1 |  |  | 3212 | -0.362 | -0.3480 | No |
| 35 | P4HA1 |  |  | 3355 | -0.382 | -0.3697 | No |
| 36 | TWSG1 |  |  | 3528 | -0.415 | -0.3967 | No |
| 37 | IFNGR1 |  |  | 3532 | -0.415 | -0.3912 | No |
| 38 | PUS1 |  |  | 3574 | -0.423 | -0.3929 | No |
| 39 | SNX9 |  |  | 3591 | -0.426 | -0.3897 | No |
| 40 | MAFF |  |  | 3620 | -0.432 | -0.3888 | No |
| 41 | GLIPR2 |  |  | 3731 | -0.450 | -0.4034 | No |
| 42 | LCLAT1 |  |  | 3764 | -0.458 | -0.4028 | No |
| 43 | LRRC8C |  |  | 3856 | -0.478 | -0.4134 | No |
| 44 | CCND3 |  |  | 3901 | -0.487 | -0.4147 | No |
| 45 | RNH1 |  |  | 3956 | -0.499 | -0.4178 | No |
| 46 | SERPINB6 |  |  | 3981 | -0.506 | -0.4150 | No |
| 47 | TNFSF10 |  |  | 4229 | -0.574 | -0.4541 | No |
| 48 | NFIL3 |  |  | 4309 | -0.600 | -0.4605 | No |
| 49 | ITGAV |  |  | 4312 | -0.601 | -0.4522 | No |
| 50 | GSTO1 |  |  | 4414 | -0.641 | -0.4622 | Yes |
| 51 | PNP |  |  | 4417 | -0.641 | -0.4532 | Yes |
| 52 | ODC1 |  |  | 4437 | -0.649 | -0.4474 | Yes |
| 53 | UCK2 |  |  | 4438 | -0.649 | -0.4379 | Yes |
| 54 | MYO1C |  |  | 4578 | -0.725 | -0.4541 | Yes |
| 55 | NT5E |  |  | 4607 | -0.737 | -0.4487 | Yes |
| 56 | PLSCR1 |  |  | 4627 | -0.752 | -0.4414 | Yes |
| 57 | SLC2A3 |  |  | 4629 | -0.754 | -0.4306 | Yes |
| 58 | F2RL2 |  |  | 4635 | -0.759 | -0.4205 | Yes |
| 59 | ARL4A |  |  | 4695 | -0.791 | -0.4203 | Yes |
| 60 | PLAGL1 |  |  | 4700 | -0.797 | -0.4094 | Yes |
| 61 | CKAP4 |  |  | 4749 | -0.828 | -0.4065 | Yes |
| 62 | SLC39A8 |  |  | 4788 | -0.850 | -0.4014 | Yes |
| 63 | CAPG |  |  | 4812 | -0.877 | -0.3931 | Yes |
| 64 | ANXA4 |  |  | 4817 | -0.882 | -0.3810 | Yes |
| 65 | SOCS2 |  |  | 4827 | -0.893 | -0.3697 | Yes |
| 66 | TGM2 |  |  | 4837 | -0.903 | -0.3582 | Yes |
| 67 | LIF |  |  | 4841 | -0.907 | -0.3455 | Yes |
| 68 | ENPP1 |  |  | 4854 | -0.918 | -0.3345 | Yes |
| 69 | MYC |  |  | 4883 | -0.947 | -0.3260 | Yes |
| 70 | KLF6 |  |  | 4953 | -1.024 | -0.3243 | Yes |
| 71 | ITIH5 |  |  | 4956 | -1.025 | -0.3098 | Yes |
| 72 | EMP1 |  |  | 4977 | -1.056 | -0.2982 | Yes |
| 73 | PIM1 |  |  | 5002 | -1.095 | -0.2868 | Yes |
| 74 | XBP1 |  |  | 5032 | -1.162 | -0.2754 | Yes |
| 75 | HOPX |  |  | 5056 | -1.204 | -0.2623 | Yes |
| 76 | PLEC |  |  | 5065 | -1.219 | -0.2460 | Yes |
| 77 | GADD45B |  |  | 5099 | -1.292 | -0.2335 | Yes |
| 78 | CDC6 |  |  | 5107 | -1.314 | -0.2157 | Yes |
| 79 | ALCAM |  |  | 5118 | -1.327 | -0.1982 | Yes |
| 80 | SOCS1 |  |  | 5138 | -1.391 | -0.1816 | Yes |
| 81 | SLC1A5 |  |  | 5154 | -1.444 | -0.1634 | Yes |
| 82 | NRP1 |  |  | 5177 | -1.553 | -0.1450 | Yes |
| 83 | SCN9A |  |  | 5207 | -1.686 | -0.1259 | Yes |
| 84 | AHNAK |  |  | 5244 | -1.992 | -0.1038 | Yes |
| 85 | PTCH1 |  |  | 5261 | -2.318 | -0.0730 | Yes |
| 86 | SPP1 |  |  | 5277 | -2.671 | -0.0369 | Yes |
| 87 | COL6A1 |  |  | 5278 | -2.689 | 0.0023 | Yes |
Table: GSEA details [plain text format]

  

Fig 2: HALLMARK\_IL2\_STAT5\_SIGNALING: Random ES distribution      
 Gene set null distribution of ES for **HALLMARK\_IL2\_STAT5\_SIGNALING**
